# Predicting the replicability of social science lab experiments

**DOI:** 10.1371/journal.pone.0225826

**Published:** 2019-12-05

**Authors:** Adam Altmejd, Anna Dreber, Eskil Forsell, Juergen Huber, Taisuke Imai, Magnus Johannesson, Michael Kirchler, Gideon Nave, Colin Camerer

**Affiliations:** 1 Department of Economics, Stockholm School of Economics, Stockholm, Sweden; 2 SOFI, Stockholm University, Stockholm, Sweden; 3 Universität Innsbruck, Innsbruck, Austria; 4 LMU Munich, Munich, Germany; 5 The Wharton School, University of Pennsylvania, Philadelphia, Pennsylvania, United States of America; 6 California Institute of Technology, Pasadena, California, United States of America; Tilburg University, NETHERLANDS

## Abstract

We measure how accurately replication of experimental results can be predicted by black-box statistical models. With data from four large-scale replication projects in experimental psychology and economics, and techniques from machine learning, we train predictive models and study which variables drive predictable replication. The models predicts binary replication with a cross-validated accuracy rate of 70% (AUC of 0.77) and estimates of relative effect sizes with a Spearman *ρ* of 0.38. The accuracy level is similar to market-aggregated beliefs of peer scientists [1, 2]. The predictive power is validated in a pre-registered out of sample test of the outcome of [3], where 71% (AUC of 0.73) of replications are predicted correctly and effect size correlations amount to *ρ* = 0.25. Basic features such as the sample and effect sizes in original papers, and whether reported effects are single-variable main effects or two-variable interactions, are predictive of successful replication. The models presented in this paper are simple tools to produce cheap, prognostic replicability metrics. These models could be useful in institutionalizing the process of evaluation of new findings and guiding resources to those direct replications that are likely to be most informative.

## 1 Introduction

Replication lies at the heart of the process by which science accumulates knowledge. The ability of other scientists to replicate an experiment or analysis demonstrates robustness, guards against false positives, puts an appropriate burden on scientists to make replication easy for others to do, and can expose the various “researcher degrees of freedom” like p-hacking or forking [4–20].

The most basic type of replication is “direct” replication, which strives to reproduce the creation or analysis of data using methods as close to those used in the original science as possible [21].

Direct replication is difficult and sometimes thankless. It requires the original scientists to be crystal clear about details of their scientific protocol, often demanding extra effort years later. Conducting a replication of other scientists’ work takes time and money, and often has less professional reward than original discovery.

Because direct replication requires scarce scientific resources, it is useful to have methods to evaluate which original findings are likely to replicate robustly or not. Moreover, implicit subjective judgments about replicability are made during many types of science evaluations. Replicability beliefs can be influential when giving advice to granting agencies and foundations on what research deserves funding, when reviewing articles which have been submitted to peer-reviewed journals, during hiring and promotion of colleagues, and in a wide range of informal “post-publication review” processes, whether at large international conferences or small kaffeeklatches.

The process of examining and possibly replicating research is long and complicated. For example, the publication of [22] resulted in a series of replications and subsequent replies [23–26]. The original findings were scrutinized in a thorough and long process that yielded a better understanding of the results and their limitations. Many more published findings would benefit from such examination. The community is in dire need of tools that can make this work more efficient. Statcheck [27] is one such framework that can automatically identify statistical errors in finished papers. In the same vein, we present here a new tool to automatically evaluate the replicability of laboratory experiments in the social sciences.

There are many potential ways to assess whether results will replicate. We propose a simple, black-box, statistical approach, which is deliberately automated in order to require little subjective peer judgment and to minimize costs. This approach leverages the hard work of several recent multi-investigator teams who performed direct replications of experiments in psychology and economics [2, 7, 28, 29]. Based on these actual replications, we fit statistical models to predict replication and analyze which objective features of studies are associated with replicability.

We have 131 direct replications in our dataset. Each can be judged categorically by whether it replicated or not, by a pre-announced binary statistical criterion. The degree of replication can also be judged on a continuous numerical scale, by the size of the effect estimated in the replication compared to the size of the effect in the original study. As binary criterion, we call replications with significant (*p* ≤ 0.05) effects in the same direction as the original study successful. For the continuous measure, we study the ratio of effect sizes, standardized to correlation coefficients. Our method uses machine learning to predict outcomes and identify the characteristics of study-replication pairs that can best explain the observed replication results [30–33].

We divide the objective features of the original experiment into two classes. The first contains the statistical design properties and outcomes: among these features we have sample size, the effect size and p-value originally measured, and whether a finding is an effect of one variable or an interaction between multiple variables. The second class is the descriptive aspects of the original study which go beyond statistics: these features include how often a published paper has been cited and the number and past success of authors, but also how subjects were compensated. Furthermore, since our model is designed to predict the outcome of specific replication attempts we also include similar properties about the replication that were known beforehand. We also include variables that characterize the difference between the original and replication experiments—such as whether they were conducted in the same country or used the same pool of subjects. See S1 Table for a complete list of variables, and S2 Table for summary statistics.

The statistical and descriptive features are objective. In addition, for a sample of 55 of the study-replication pairs we also have measures of subjective beliefs of peer scientists about how likely a replication attempt was to result in a categorical Yes/No replication, on a 0-100% scale, based on survey responses and prediction market prices [1, 2]. Market participants in these studies predicted replication with an accuracy of 65.5% (assuming that market prices reflect replication probabilities [34] and using a decision threshold of 0.5).

Our proposed model should be seen as a proof-of-concept. It is fitted on an arguably too small data set with an indiscriminately selected feature set. Still, its performance is on par with the predictions of professionals, hinting at a promising future for the use of statistical tools in the evaluation of replicability.

## Materials and methods

The data are combined from four replication projects, The Reproducibility Project in Psychology (RPP; [7]), the Experimental Economics Replication Project (EERP; [2]) and Many Labs (ML) 1 and 3 [28, 29]. In most cases, one specific statistical test from each paper was selected for replication, but four papers had multiple effects replicated. In RPP and EERP, each experiment was replicated once. In the Many Labs projects all participating labs replicated every experiment and the final results were calculated from the pooled data. A total of 144 effects were studied (100 RPP experiments, 16 from ML1, 10 from ML3, 18 from EERP). After dropping observations with missing values, our final data set contains 131 study-replication pairs. For 55 of these observations we also have data on prediction markets prices [1, 2] that we use as a benchmark when we evaluate the model.

### 1.1 Dependent variables

There is no single best replication success indicator. An active literature studies different strategies to evaluate replicability (see e.g. [35, 36]). For this paper, we have chosen to prioritize simplicity and focus on two measures, one binary and one continuous:

$$\begin{array}{cc} \text{Replicated} & =\{\begin{array}{cc} 1 & p_{\text{replication}}\leq 0.05\;\text{and}\;\text{effect}\;\text{in}\;\text{same}\;\text{direction}\\ 0 & \text{otherwise} \end{array}\\ \text{Relative}\;\text{Effect}\;\text{Size}\;\text{Estimate} & =\frac{\text{replication}\;\text{effect}\;\text{size}\;\text{(}r\text{)}}{\text{original}\;\text{effect}\;\text{size}\;\text{(}r\text{)}} \end{array}$$

**Fig 1:**
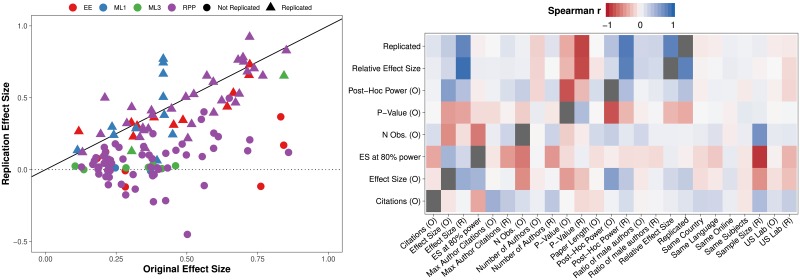
Effect sizes and correlations. (A) A plot of effect sizes (*r*) in each study pair. Data source is coded by color. Symbol shape denotes whether a study replicated (binary measure). Most points are below the 45-degree line, indicating that effect sizes are smaller in replications. Replications with a negative effect size have effects in the opposite direction compared to the original study. (B) A heatmap showing Spearman rank-order correlations between variables. Y-axis shows most important features with the two dependent variables on the top. O and R in the variable label correspond to original and replication studies, respectively. Plus and minus indicate positive and negative correlations respectively. Most correlations are weak. See S1 Table for variable definitions and S1 Fig for a full correlation plot.

The binary model defines replication success as a statistically significant (*p* ≤ 0.05) effect in the same direction as in the original study. This measure has often been criticized and is indeed simplistic. We use it since it can be compared to prediction market estimates from previous studies (where subjects traded bets using the same measure). According to this definition, 56 replications are successful and 75 fail. 22 replication had effects going in the opposite direction compared to the original, but all with p-values larger than 0.05. The continuous model predicts a ratio between two estimates of the effect size, from the replication and original study respectively, both standardized to correlation coefficients. It yields a more fine-grained notion of replication that does not depend on the peculiarities of hypothesis testing. In our data, the relative effect size varies between −0.9 and 2.38 with a mean of 0.49. As can be seen in the left plot of Fig 1, most relative effect size estimates close to 0 are also “unsuccessful replications” in terms of the binary metric.

### 1.2 Independent variables

For each original-replication pair, we have collected a large set of variables (see Fig 1B for the variable names or S1 Table for descriptions). The feature set includes objective characteristics of the original experiment, but also information about the replication that was known *before* it was carried out. For example, these variables include statistical information such as the standardized effect size and p-value of the original experiment, but also contextual information such as the type of compensation used, the highest seniority and gender composition of the replication team, as well the length of the paper are included. Note that the standardized original effect size is included in the continuous model even though it is also the denominator of the outcome variable. The model can therefore be thought of as predicting the change in estimated standardized effect size between the original study and it’s replication.

The only transformations we have included are commonly used statistical variables (power, standardized effect size and p-value are all non-linear transforms of each other), but we decided against the inclusion of any other transformations as it would increase the feature space too much. Some such transforms (like log citations) would probably help the linear models in our comparisons. Since the model we end up using is non-linear however, it should not matter much for final performance.

We intentionally provide no theoretical justification for the inclusion of any specific feature, but have simply gathered as many variables as possible. We leave it to the user of the model to decide which of these variables are relevant for their specific implementation, and provide information about the relative importance of each feature.

The heatmap of Spearman rank-order correlation coefficients in Fig 1B shows some correlation between our two outcomes and other features (the top two rows). Most relationships are weak. Ex-ante expected correlations are strong (e.g., sample size and p-value) but not many other relationships are evident visually (see S1 Fig for a full correlation plot). Original effect sizes are correlated with binary replication and so are p-values, with Spearman *ρ* of 0.26 and 0.38, respectively.

### 1.3 Model training

**Fig 2:**
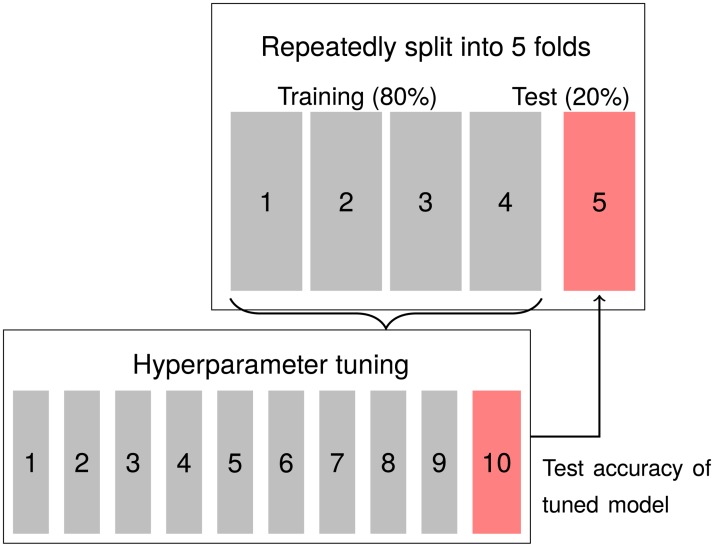
Model training, nested cross-validation (CV). First, the data is split into five parts. Four parts are used for training. For each model a 10-fold CV is run on this training data to find optimal hyperparameters for each algorithm. When training the LASSO, different values for λ (penalty to weakly correlated variables) are tested, for Random Forest the number of randomly selected features to consider at each split changes. In each run the model is trained on 9/10th of the data and tested on the last decile. The best version (with highest AUC) is trained on all of the training data and its accuracy is estimated on the fifth fold of the outer loop. The process is repeated with a different outer fold held out. After five runs, a new set is drawn, and the process is repeated until 100 accuracy metrics have been generated.

**Fig 3:**
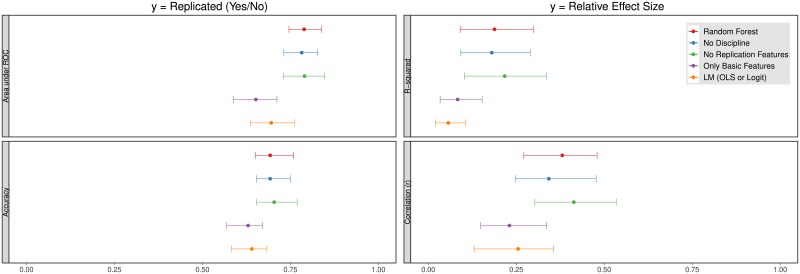
Interquartile range (IQR) and median of Random Forest classifier (left) and regression (right) validation set performance. For the classifier, the optimal model (first from top) has an average AUC of 0.79 and accuracy of 70% at the 50% probability cutoff (accuracy is mainly driven by a high true negative rate; unsuccessful replications are predicted with an accuracy of 80%, while successful only with 56%). The optimal regression model has a median *R*^2^ of 0.19 and a Spearman *ρ* of 0.38. The second bar from the top in each subplot shows unchanged model performance when dummy indicators for discipline (Economics, Social or Cognitive Psychology) are removed. The third has excluded any features unique to the replication effort (e.g. replication team seniority) with no observable loss of performance. The less accurate fourth model is only based on original effect size and p-value. Last, the model at the bottom is a linear model trained on the full feature set, for reference. See S3 Fig for more models.

We use cross-validation to avoid overfitting. To simultaneously evaluate variability of the accuracy metric we nest two cross-validation loops, as shown in Fig 2. In the inner loop, we search and validate algorithm-specific hyperparameters. Each such optimally configured model is then tested on 20% of the data in the outer loop. Our limited sample size forces us to use these validation sets for both reported performance statistics and algorithm selection (see Fig 3 and S3 Fig). Because we make decisions based on these performance statistics, also our cross-validated measures may suffer from some overfitting. We therefore evaluate pre-registered predictions of the results of [3] as a supplement.

When training the binary classification models, we do so with the goal to maximize the area under the curve (AUC) of a receiver operating characteristics (ROC) curve [37]. The metric accounts for the trade-off between successfully predicting positive and negative results respectively. Maximizing accuracy might result in a model that always predicts experiments to not replicate, and thus accurately predicts all unsuccessful replications, but incorrectly classifies all those that do replicate. The model with the highest AUC will instead be the one that minimizes the effects of this trade off, achieving high prediction rates for both positive and negative results simultaneously. The models estimating relative effect size are trained to minimize the mean squared prediction error.

We compare a number of popular machine learning algorithms (see S3 Fig) and find that a Random Forest (RF) model has the highest performance. The outcome predicted by an RF algorithm is the result of averaging over a “forest” of decision trees. Each tree is fitted using a random subset of variables, and employs a hierarchical sequence of cutoffs to predict observations [38]. A simple tree with depth 2 might fit 0-1 replication success by first dividing cases by if sample size is below a cutoff, then, at each of those two branches, by whether the original effect is a main effect or an interaction. Each end node is a prediction of the outcome variable. The algorithm is popular because it performs well without much human supervision.

## 2 Results

The Random Forest model trained on the full feature set predicts binary replication in the hold-out sample with a median AUC of 0.79 (median accuracy of 69% at the 50% probability threshold), shown in the top bars of Fig 3. The bar width is the interquartile range of 100 performance resamples produced by the nested cross-validation. The median is depicted as a dot.

Note that we are predicting the outcome of a statistical test, an inherently noisy variable. The upper bound for ideal replication forecasting is thus less than 100%; probably also below 90%. Why? Consider an artificial sample, measuring a, by construction, genuine effect with tests that have 90% power to detect it. A perfect model predicting replication in a second sample will only be right nine out of ten times. This theoretical ceiling is important since we should arguably normalize the distance between random guessing and the best possible level of prediction. For 69% accuracy, the normalized improvement over a random guess (50%) to perfection is $\frac{69-50}{100-50}=0.38$. However using a more accurate upper bound of 90%, it is $\frac{69-50}{90-50}=0.48$.

The model predicting the Relative Effect Size estimates achieves a median root mean squared error (RMSE) of 0.51 and *R*^2^ of 0.19. The predicted and actual effect sizes have a median Spearman correlation of 0.36. It is important to note that similarly to the binary replication indicator, perfect prediction of the relative effect size is not possible, because the outcome variable is the ratio of noisy estimates of effect sizes. The lower bound of the prediction error in this case depends on the variance of the effect size measurement in the original study and the replication, as well as the covariance between them. Deriving a theoretical upper bound for the measure is beyond the scope of the current work, but see S2 Text for a discussion.

In the pre-registered out of sample test, 71% of binary replications are predicted correctly (AUC: 0.72). Relative effect size is estimated with a RMSE of 0.41 (Spearman *ρ*: 0.25; *R*^2^: 0.07).

A qualitative assessment of these results can be made in both relative and absolute terms.

First, binary classifier performance is substantially higher than that of a random model (which by definition has an AUC of 0.5), and is more accurate than a linear model using the same features (the last bar in each subplot of Fig 3, median AUC = 0.72). A constant model that never predicts a paper to replicate would be far worse, with an accuracy of 57% in the training set.

The continuous model does not perform as well. Considering that the relative effect size estimates range from −0.9 to 2.38, an RMSE of 0.51 is a substantial error. The range is smaller in the out of sample test set (−0.12 to 1.3), something that could explain the smaller error of 0.41. While also the linear relationship and Spearman correlation are weak, an OLS regression performs even worse in the training set, with an *R*^2^ of 0.06. The Spearman *ρ* of 0.36 (OLS: 0.27) between predicted and actual values is higher than the 0.21 correlation between original and relative effect size estimate, indicating a performance improvement over this very simple heuristic.

When performance is compared between validation set and the pre-registered test set, the binary classifier achieves similar results. The continuous model, however, manages to explain only 7% of the variation and has a Spearman correlation coefficient that is 30% smaller. Such large differences between validation and test sets could be an indication of overfitting.

Second, the predictions of the binary classifier, based on objective features, can be compared to subjective beliefs of replicability generated from prediction market prices. We get these beliefs from earlier studies where social scientists traded on the probability of replication success. Participants in these studies had access to both the original papers and pre-registered replication plans, describing how the replications were going to be conducted. Participants did not know the estimated replication probabilities, but could in theory have trained a model themselves. In other words, all the features that were used in the model were in principle also available to market participants. Out of the 55 replications we have both model and market predictions for, the market predicted 65.5% correctly (accuracy was 68% for studies in [1] and 61% in [2]). While the model fares slightly better in this data, two follow-up papers have more accurate markets. Including market performance from [3, 39] yields a prediction market accuracy rate of 73% (76/104 replications) (with an AUC of 0.73).

### 2.1 Predictive power

In Fig 3, we compare the performance of models in which certain classes of variables have been excluded. The observation of similar patterns for both sets of models is not surprising, given the high correlation of the two outcome measures (Spearman *ρ* = 0.79). The predictions of relative effect size estimates are much noisier however, hinting at the general inaccuracy of the model.

For both replication measures, the second bar shows that removing the dummy variables encoding the discipline of the study (Economics, Social Psychology or Cognitive Psychology) has little bearing on the results. The 64 Social Psychology replications have smaller effect sizes (mean of 0.33 compared to 0.47 for cognitive psychology), slightly larger p-values (0.017 compared to 0.01). In [40], the author argues that the association between contextual sensitivity (as measured on a scale from 0-5) and replicability found by [8] is spuriously identified from the difference in replication rates between fields. We show that many other variables also mediate these differences. For example, by construction, holding sample size constant, interaction effects will have lower statistical power. Included social psychology experiments test interaction effects almost twice as often (44% vs 27% in cognitive psychology). If studies of interactions do not increase sample size appropriately, replicability will be lower.

The third bar shows no reduction in accuracy for a model in which all replication-specific features are excluded. The reason is likely that replication characteristics were standardized between experiments. No replication is conducted with a really small sample size, for example.

The fourth bar uses only original effect size and p-value. The decrease in accuracy shown in this bar implies that also other features are informative.

### 2.2 Feature importance

The previous section summarized the *general* accuracy of the models, using different feature subsets. This section explores which objective features of experimental designs and results are important for replicability, extending the analysis in RPP [7] with more variables, non-linear interactions, and a larger data set.

**Fig 4:**
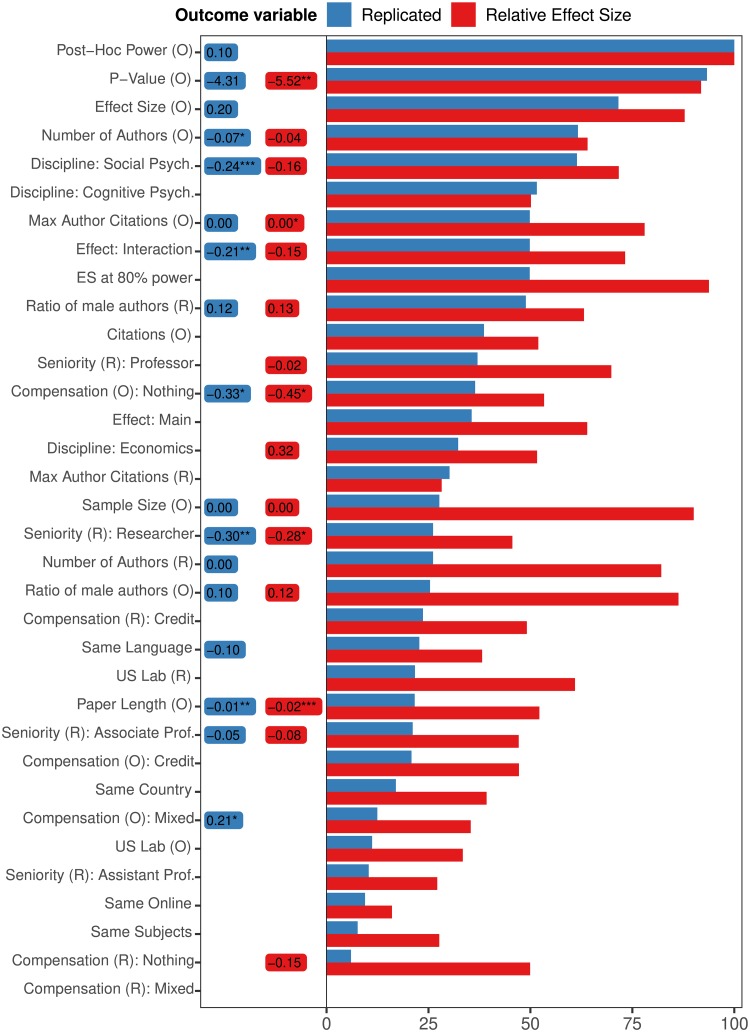
Right side contains relative variable importance for all features used in the Random Forest, for both regression (red) and classification (blue) models, sorted by decreasing contribution to the predictive power of the binary classifier. To the left are average marginal effects for those variables selected by a LASSO and then re-fit in a linear model (Logit for binary, OLS for continuous). Predictably, most of the top variables are statistical properties related to replicability and publication, but also other variables seem to be informative, especially for the Random Forest. For example, whether or not the effect tested is an interaction effect, as well as the number of citations are important. Last, note that the two top variables are basically non-linear transformations of one another. Stars indicate significance: *p* ≤ 0.01(***), *p* ≤ 0.05(**), and *p* ≤ 0.1(*).

The action-packed Fig 4 reports two metrics of feature importance for both the binary (blue) and continuous (red) models. The length of horizontal bars (x-axis) represents Random Forest variable importance, measured as the relative frequency at which features are selected in individual decision trees. Features that are included in a large proportion of the individual trees will have a long bar. The variables are sorted by their importance in predicting binary replication. The three most important variables are post-hoc power, p-value, and effect size of the original studies. They are the same for both the binary and continuous model. Since effect size is standardized, all three of these variables are actually non-linear transformations of each other.

Since the RF model is hierarchical and nonlinear, a single variable can be included in many different individual trees with both positive and negative effects on predicted outcome. While we can identify the most important variables in the model, we cannot determine the direction of their influence. We therefor also present the average marginal effect of each variable in linear models (Logit for binary, OLS for continuous). These are shown in small boxes between the variable names (on the left) and the bars on the right. This analysis uses only variables that have been selected as important by a LASSO, a regularization algorithm, that minimizes squared errors (or deviance) while keeping the absolute value of coefficients constrained by a penalty term. This method tends to shrink estimated coefficients that are unimportant towards zero, removing some variables completely [31]. For the many variables that are common in the RF trees but have zero LASSO weights, there are blank spaces between variable definitions and RF-frequency bars. The features selected by the LASSO are then re-fitted in a regular Logit model (to “unshrink” their weights) and the coefficients of that non-regularized model are presented Fig 4. Note that there is no clear mapping beteween the two measures of importance. Logit estimates describe the average linear relationship between the coefficients and the log odds of replication. A variable could have a positive linear estimate while the RF assigns a negative relationship for almost all cases.

### 2.3 Pre-registered out of sample test

**Fig 5:**
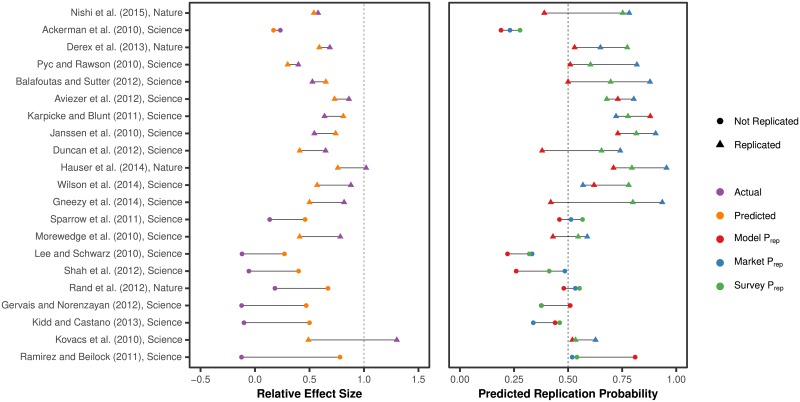
Predicted and actual results of the SSRP. Model predictions were registered before the experiments had been conducted. The left panel shows predicted relative effect size in purple and actual in orange, sorted by increasing prediction error. Right panel shows replication probability as predicted by the model, a prediction market, and a survey respectively. Data points are represented by a triangle when the replication was successful (*p* < 0.05 and an effect in the same direction). To see when the model made a correct prediction at the 50% probability threshold study the right panel. Red triangles on the right side of the dashed line and circles on the left side have been predicted correctly.

The Social Sciences Replication Project (SSRP; [3]) replicated 21 systematically selected papers published in *Nature* and *Science* published between 2011 and 2015. The authors also collected beliefs through a survey and a prediction market. We registered the predictions of the model before the replications had been conducted [41]. The results from this out of sample test are summarized and compared to market and survey beliefs in Fig 5. The replications were conducted in a two-stage procedure, where more data was collected if the results from the first phase were not significant. Here, we use the results from the pooled data. If the model predicted a successful Stage 1 replication these predictions are used. If it predicted an unsuccsessful first stage, predicted effect size and replication probability from Stage 2 are used instead. In S4 Fig we test predictions on Stage 1 outcomes. The results are similar.

The out of sample predictions achieve accuracy similar to the median cross-validated level, at 71% (AUC: 0.73). When compared to researcher beliefs, the model has a mean absolute prediction error of 0.43, while the market achieves 0.30 and the survey 0.35. The difference between model and market is significant (Wilcoxon signed-rank test, *z* = −2.52, *p* = 0.012, *n* = 21), however more data is needed to verify these differences.

The model predicts relative effect size estimates with a Spearman correlation of 0.25 (*p* = 0.274), lower than the cross-validated measure of 0.38. The mean absolute deviation is 0.33. A Wilcoxon sign-rank test cannot reject that the distributions of predicted and actual effect sizes are the same, *z* = −1.00, *p* = 0.317.

Results are summarized in Fig 5. We see that the model produces quite conservative forecasts of effect size, often closer to 0.5 than the actual outcome. This results in large errors whenever the actual effect is substantially different from half the original effect. This leads to especially poor predictions of relative effect size estimates for unsuccessful replications. While the market and survey perform better than the model in predicting binary replication in this sample, the plot shows how the measures commonly yield the same prediction. When they do not, it is often because the model incorrectly predicts that an experiment will not replicate.

## 3 Discussion

We have derived an automated, data-driven method for predicting replicability of experiments. The method uses machine learning to discover which features of studies predict the strength of actual replications. Even with our fairly small data set, the model can forecast replication results with substantial accuracy—around 70%.

Predictive accuracy depends on the features of the model in interesting ways. The statistical properties (p-value and effect size) of the original experiment are the most predictive. However, the accuracy of the model increases when variables such as the nature of the finding (an interaction, compared to a main effect), number of authors, paper length, and the lack of performance incentives are added. All those variables are linearly associated with a reduction in the predicted chance of replicability.

The third bar in Fig 3 shows unchanged performance for a model with all replication-specific features excluded. There are a couple of possible reasons why removing replication features has no impact on model performance. For one thing, most variables have a small impact, which would be easier to identify in a larger data set. Second, a larger planned sample size has a direct impact on replication probability, since with higher power it follows that there is a higher probability of rejecting a false null hypothesis, and thus also the corresponding probability of replicating a true result (See e.g. [42] for a discussion of replication power). The reason why we do not find such a relationship is probably because our data has little variation in power, as most replications are designed to have larger samples, and do not include multiple replications of the same experiment with different sample sizes. This makes it hard for the model to capture any variation in replicability caused by changes in planned sample size. It is also possible that the model is unable to separate the increase in power from the fact that weaker effects required larger replication samples.

The fourth bar in Fig 3 presents the accuracy of a simple model that is only trained on effect size and p-value of the original experiment. It is not quite as accurate as models with more features, but still on par with the linear model trained on the full feature set. The analysis of correlations in [7] indicated the opposite, that experience of the experimenters and other such features are unimportant. With the substantial variability in out-of-sample accuracy, it is difficult to say for sure, but our results do indicate that these other features are correlated and indeed contribute to higher accuracy.

We now probe a bit further into three results.

The first result is that one variable that is predictive of poor replicability is whether central tests describe interactions between variables or (single-variable) main effects. Only eight of 41 interaction effect studies replicated, while 48 of the 90 other studies did. As Fig 4 shows, the interaction/main effect variable is in the top 10 in RF importance and is predictive, for both the binary and continuous replication measures.

There is plenty of room for reasoned debate about the validity of apparent interactions. Here is our view: Interactions are often slippery statistically because detecting them is undermined by measurement error in either of two variables. In early discussion of p-hacking it was also noted that studies which hoped to find a main effect often end up concluding that there is a main effect which is only significant in part of the data (i.e., an interaction effect). The lower replication rates for interaction effects might be spurious, however. Tests of interactions often require larger samples, which could mean that the replications of these studies have lower power relative to those studies evaluating non-interacted effects. Nonetheless, the replicability difference is striking and merits further study. It is possible that the higher standard of evidence for establishing interactions needs to be upheld more closely.

The second result is that some features that vary across studies are *not* robustly associated with poor replication: These include measures of language, location and subject type differences between replication and original experiments, as well as most of the variation in compensation (except for having no compensation at all, which is correlated with lower replicability).

Our third result is that the model performs on par with previously collected peer judgments (subjective beliefs as measured by prediction market prices). In the sample used to estimate the model, it performed somewhat better than the prediction market, although we only had prediction market data on a subset of *n* = 55 studies. On the other hand the prediction market performed better than the model on the out of sample prediction test, but this was based on a small sample of *n* = 21. More data is needed to compare statistical approaches with peer judgments in prediction markets and surveys, to test which approach is associated with the most accurate predictions, and to look for potential complementarities. If the goal is replication prediction, the model has logistical advantages compared to running prediction markets, which require both participants and costly monetary incentives.

Studying the differences between our algorithmic predictions and expert scientific judgment adds to a long literature comparing machine and man. For at least seventy years, it has been known that in many domains of professional judgment, simple statistical models can predict complex outcomes—PhD success, psychiatric disorders, recidivism, personality—as accurately as humans do subjectively [43–46]. Today, with the tremendous increase in data availability and development of more sophisticated predictive models, statistical prediction has become useful in many new areas (e.g. [47]). It is likely that in some form, statistical methods will also increase the quality of human evaluation and prediction of scientific findings. The results presented in this paper suggests that there could be room for statistical methods to aid researchers when reviewing their peers’ experiments. An interesting avenue for future research is to look for potential synergies. Do market participants who get access to model predictions perform better? Does including market prices as a feature improve model performance?

### 3.1 Applications

Our method could be used in pre- and post-publication assessment, preferably after a lot more replication evidence is available to train the algorithm. In the current mainstream pre-publication review process, the decision about whether to publish a paper is almost entirely guided by the opinions of a small set of peer scientists and an editor. A systematic, fast, and accurate numerical method to estimate replicability could add more information in a transparent and fair way to this process. For example, when a paper is submitted an editorial assistant can code the features of the paper, plug those features into the models, and derive a predicted replication probability. This number could be used as one of many inputs helping editors and reviewers to decide whether a replication should be conducted before the paper is published.

Post-publication, the model could be used as an input to decide which previously published experiments should be replicated. The criteria should depend on the goal of replication efforts. If the goal is to quickly locate papers unlikely to replicate, then papers with low predicted replicability should be chosen. Since replication is costly and laborious, using predicted probability can guide scarce resources toward where they are most scientifically useful.

**Fig 6:**
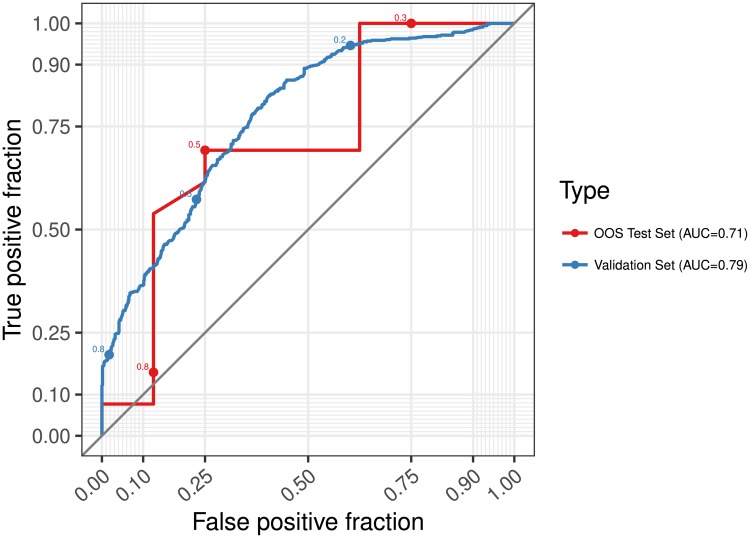
ROC curve for held-out validation sets from the best model during cross-validation and for the out of sample predictions. The plot shows the trade off between true positives (predicting correctly that a study will replicate) and false positives (predicting that a study will replicate when it in fact does not) as the decision threshold varies. At a threshold of 0.5 the model identifies about 70% of the successful replications and 75% of the non-replications correctly. If a user of the model wants to lower the risk of missclassifying a paper that would replicate as not replicating they can use a threshold of e.g. 0.3. At this level, the model misclassifies less than 10% of the successful replications. The price, however, is that almost 70% of non-replications will also be labeled as successful.

Choosing an appropriate decision threshold is an important part of applying models such as ours in practice. The cost of carrying out additional replication may vary between studies, and so does the cost of publishing a false positive finding. For example, an editor could require original authors to run a replication whenever the replication probability of their submission is below 0.7. As can be seen in the receiver operating curve (ROC) plotted in Fig 6, such a threshold would ensure that only 10% of non-replicable results would pass through undetected. Had the editor used a threshold of 0.5 (like we do in this paper to calculate accuracy) 25% of the predictions about successful replication would be incorrect, but fewer (∼30%) unnecessary replications would be carried out. Moreover, changes to the machinery of the algorithm could be introduced in order to optimize for specific trade offs between true and false positives. We optimize AUC and leave the choice of threshold to the user of the model. But another alternative is to optimize with asymmetric costs pre-assigned to different types of errors akin to the method of Masnadi-Shirazi & Vasconcelos [48]. We encourage any user to think carefully about this decision rule, as the relative cost of making positive and negative prediction errors might vary greatly between applications.

An important concern with any predictive algorithm is that its application will likely change incentives, and impact how scientists design their studies, undermining the algorithm’s value. Some of these “corrupting” [49] effects will actually be good: For example, since testing interaction effects seem to negatively associated with predicted replicability, scientists may be motivated to avoid searching for such interactions. But that could be an improvement, if such effects are difficult to find robustly with sample sizes used previously. Alternatively, scientists who are keen to find interactions can use higher-powered designs, which will increase predicted replicability.

Other changes in practice to “game” the algorithm will likely be harmless, and some changes could reduce predictive accuracy. For example, longer papers tend to replicate less well. If scientists all shorten their papers (to increase their predicted replicability), without changing the quality of the science, then the paper length variable will gradually lose diagnostic value. Any implementation will need to anticipate this type of gaming.

Some types of “gaming” could be truly unwanted. The trade off between algorithmic fairness and accuracy is a highly important question that is currently being studied extensively. In our case, including the gender composition or seniority of the author team potentially increases the risk that the model is discriminatory. If needed, such variables could easily be removed, with only a small penalty to accuracy. However, excluding a variable like gender composition will not necessarily remove the model’s tendency to discriminate, as this variation could still be captured through other features [50]. We included these variables here to make this trade-off transparent.

Of course, there are limits to how much we can conclude from our results. The data we use is not representative for all experimental social science—the accuracy level and variable importance statistics may be specific to our sample, or to psychology and economics. Our sample of studies is also very small; having more actual replications, preferably selected randomly, is crucial to ensure that the model functions robustly [51].

Moreover, the correlations we find do not identify causal mechanisms, so changing research practices (as in the “gaming” scenarios above) may have unknown consequences. Rather, our model is theory agnostic by design. We aim to predict replicability, not understand its causes. The promising and growing literature taking a theoretical approach to this questions (see e.g. [4, 35, 36]) should be seen as a complement to our work and could hopefully be used to improve future versions of this predictive model. Simultaneously, our insights will hopefully be useful for future theoretical investigations.

The future is bright. There will be rapid accumulation of more replication data, more outlets for publishing replications [52], new statistical techniques, and—most importantly—enthusiasm for improving replicability among funding agencies, scientists, and journals. An exciting replicability “upgrade” in science, while perhaps overdue, is taking place.

## Supporting information

S1 Table Variable descriptions. Explanations of all variables in the data set.
(PDF)

S2 Table Summary statistics. Summary statistics for all variables in the data set, divided into tables of continuous, binary, and categorical variables respectively.
(PDF)

S3 Table Prediction market accuracy. Accuracy of prediction market data used in the paper. The ending prices for each asset are directly interpreted as replication probabilities and prediction accuracy is calculated based on a 50% probability cutoff.
(PDF)

S4 Table PCA—Explained variance. The contribution of all 15 principal components. Contributed variance tapers off quite slowly, indicating the lack of any strong linear structure.
(PDF)

S1 Text Data management and decisions. (PDF)

S2 Text Predicting a noisy outcome. Discussion about the theoretical upper bound of prediction accuracy.
(PDF)

S1 Fig Full correlation plot. Plot of Spearman correlations between all pairs of variables, the full version of Fig 1.
(EPS)

S2 Fig PCA plot. The two most important principal components that together explain 40% of the variation in the continuous features of the data. It seems like the second component captures differences in statistical variables such as p-value and effect size, while the first explains author statistics.
(EPS)

S3 Fig Performance comparison of algorithms. Cross-validated hold-out sample performance of different machine learning algorithms. For each model, the outer CV loop is run 100 times. Bands show interquartile range (IQR) and the dot is the median. The left panel shows (binary) classification models, and the right includes the continuous outcomes. For each model, we tune its hyperparameters to maximize the AUC and RMSE for the binary and continuous outcome measures respectively. For Random Forest, we evaluate how many variable to randomly sample as candidates at each split and use a fixed number of trees (1001). For LASSO we tune the shrinkage parameter λ, with a fixed *σ* at 0.02 and let the cost parameter *C* vary. Last, for GBM, we vary the number of splits performed in each tree. The Random Forest model is chosen for further analysis.
(EPS)

S4 Fig SSRP prediction evaluation—Stage 1. A copy of Fig 5 but using the outcomes from the first data collection stage instead of the pooled data. The SSRP had a two-stage design. For replications that were found to be not significant in an initial test, more data was collected. Sample sizes are thus smaller for those studies that continued to a second data collection. The model is not very good at capturing this mechanical increase in replication probability from increase sample size. For one thing, we do not have any within-study variation in sample size in the data. While we could use the disaggregated Many Labs data to research within-study variation, the sample sizes do not differ much between labs.
(EPS)
