# Supplementary material for: Predicting the replicability of social science lab experiments
Source: PLoS One. 2019 Dec 5;14(12):e0225826. doi: 10.1371/journal.pone.0225826 (PMC6894796; doi:10.1371/journal.pone.0225826)
Supplement: S1 Table — Explanations of all variables in the data set. (PDF) [file pone.0225826.s001.pdf]

Table 1: Variable Descriptions

Variable	Description
Avg. Author Citations (O)	Average nr of citation of authors in original study
Avg. Author Citations (R)	Average nr of citation of authors in replication
Max Author Citations (O)	Nr of citation of the author in the original study with the highest citation count
Max Author Citations (R)	Nr of citation of the author in the original study with the highest citation count
Authors (R)	Names of replication authors
Ratio of male authors (O)	Ratio of male authors in original study
Ratio of male authors (R)	Ratio of male authors in replication
Citations (O)	Number of citations of original paper
Compensation (O)	Compensation in original experiment. One of the following: nothing, cash, credit, mixed
Compensation (O): Cash	Subjects were compensated with cash in original experiment
Compensation (O): Credit	Subjects were compensated with course credits in original experiment
Compensation (O): Mixed	Subject were compensated with both cash and credits in original experiment.
Compensation (O): Nothing	No compensation in original experiment
Compensation (R)	Compensation in replication. One of the following: nothing, cash, credit, mixed
Compensation (R): Cash	Subjects were compensated with cash in replication
Compensation (R): Credit	Subjects were compensated with course credits in replication
Compensation (R): Mixed	Subject were compensated with both cash and credits in replication.
Compensation (R): Nothing	No compensation in replication
Discipline	Discipline of original paper. One of the following: social, cognitive, economics.
Discipline: Cognitive Psych.	Cognitive Psychology
Discipline: Economics	Economics
Discipline: Social Psych.	Social Psychology
Effect Size (O)	Standardized effect size of original paper
Effect Size (R)	Standardized effect size of replication
Effect Type	Type of effect tested. One of the following: main effect, correlation, interaction
Effect: Correlation	Experiment tests a correlation effect
Effect: Interaction	Experiment tests an interaction effect
Effect: Main	Experiment tests a main effect
Prediction Market Price	Final market price in prediction market
ES at 80% power	Standardized effect size required in replication to achieve 80% power.
Experiment Country (O)	Country where original experiment was conducted
Experiment Country (R)	Country where replication is to be conducted
Experiment Language (O)	Language used with subjects in original experiment
Experiment Language (R)	Language to be used with subjects in replication
id	Unique id for each O/R pair
Replication Lab ID	Unique id for each replication lab
Paper Length (O)	Number of pages of original paper
Sample Size (O)	Sample size of original paper
Sample Size (R)	Sample size of replication
Number of Authors (O)	Number of authors in original study
Number of Authors (R)	Number of authors in replication
Planned Sample Size (R)	Planned sample size of replication
Online (O)	If the original experiment was conducted online
Online (R)	If the replication was conducted online
P-Value (O)	P-value of original paper
P-Value (R)	P-value of replication
Post-Hoc Power (O)	Post hoc power based on original effect size
Post-Hoc Power (R)	Post hoc power based on replication effect size
Planned Power (R)	Planned power of replication based on planned N and original ES
Replication Project	The replication project that the study was in. One of either: EE, RPP, ML1, ML3
Relative Effect Size	The continuous outcome variable; the standardized replication effect size relative to the original effect.
Replicated	Binary outcome variable, study is replicated if p<=0.05 and effect goes in the same direction as the original.
Same Country	Original study and replication are in the same country

Same Language	Original study and replication are in the same language
Same Online	Original study and replication are both conducted online
Same Subjects	Original study and replication use same type of subjects
Highest Author Seniority (O)	Most senior author in original paper (Professor, Associate Prof., ...)
Seniority (O): Assistant Prof.	Highest seniority in original study is Assistant Professor (rank 3/4)
Seniority (O): Associate Prof.	Highest seniority in original study is Associate Professor (rank 2/4)
Seniority (O): Professor	Highest seniority in original study is Professor (rank 1/4)
Seniority (O): Researcher	Highest seniority in original study is Researcher (rank 4/4)
Highest Author Seniority (R)	Most senior author in original paper (Professor, Associate Prof., ...)
Seniority (R): Assistant Prof.	Highest seniority in replication is Assistant Professor (rank 3/4)
Seniority (R): Associate Prof.	Highest seniority in replication is Associate Professor (rank 2/4)
Seniority (R): Professor	Highest seniority in replication is Professor (rank 1/4)
Seniority (R): Researcher	Highest seniority in replication is Researcher (rank 4/4)
Type of subjects (O)	Type of subjects in original experiment. One of the following: students, community, anyone, online
Type of subjects (R)	Type of subjects in replication. One of the following: students, community, anyone, online
PM Trading Volume	Total volume of traded stocks in prediction market
Number of PM transactions	Number of transactions in prediction market
US Lab (O)	Original experiment lab in the US
US Lab (R)	Replication experiment lab in the US
