# Supplementary material for: Predicting the replicability of social science lab experiments
Source: PLoS One. 2019 Dec 5;14(12):e0225826. doi: 10.1371/journal.pone.0225826 (PMC6894796; doi:10.1371/journal.pone.0225826)
Supplement: S2 Table — Summary statistics for all variables in the data set, divided into tables of continuous, binary, and categorical variables respectively. (PDF) [file pone.0225826.s002.pdf]

Table 2: Summary Statistics (continuous variables)

Variable	Min.	Median	Mean	Max.	NA's
author_citations_avg.o	40.00	1733.67	4210.25	44075.00	0.00
author_citations_avg.r	18.50	361.33	754.66	6063.00	0.00
author_citations_max.o	54.00	3266.00	8152.51	45135.00	0.00
author_citations_max.r	35.00	622.00	3320.81	18185.00	0.00
authors_male.o	0.00	0.67	0.71	1.00	0.00
authors_male.r	0.00	0.52	0.56	1.00	0.00
citations	6.00	72.00	264.35	13010.00	0.00
effect_size.o	0.10	0.38	0.41	0.86	0.00
effect_size.r	-0.45	0.15	0.22	0.92	0.00
endprice	0.13	0.68	0.63	0.94	76.00
es_80power	0.04	0.28	0.30	0.95	0.00
length	1.00	10.00	13.17	45.00	0.00
n_authors.o	1.00	3.00	2.83	9.00	0.00
n_authors.r	1.00	2.00	12.00	64.00	0.00
n_planned.r	5.00	100.00	922.87	6336.00	0.00
n.o	7.00	72.00	92.96	660.00	0.00
n.r	7.00	124.00	899.14	6336.00	0.00
p_value.o	0.00	0.01	0.01	0.10	0.00
p_value.r	0.00	0.14	0.26	0.98	0.00
power_planned.r	0.07	0.93	0.86	1.00	0.00
power.o	0.29	0.77	0.76	1.00	0.00
power.r	0.05	0.30	0.48	1.00	0.00
pub_year	1973.00	2008.00	2007.16	2015.00	0.00
relative_es	-0.90	0.43	0.49	2.38	0.00
trading_volume	168.96	777.51	982.58	2849.37	75.00
transactions	28.00	60.00	74.96	193.00	75.00

Table 3: Summary Statistics (binary variables)

Variable	0	1
online.o	129	2
online.r	122	9
replicated	75	56
same_country	67	64
same_language	41	90
same_online	9	122
same_subjects	18	113
us_lab.o	45	86
us_lab.r	53	78

Table 4: Summary Statistics: (discipline)

Freq	
Cognitive	49
Economics	18
Social	64

Table 5: Summary Statistics: (effect_type)

Freq	
correlation	6
interaction	41
main effect	84

Table 6: Summary Statistics: (experiment_country)

Freq	(o)	(r)
Australia	2	1
Austria	1	6
Belgium	1	0
Canada	4	3
France	4	0
Germany	10	16
Hong Kong	0	1
Israel	4	1
Italy	2	3
Netherlands	4	8
Poland	1	0
Singapore	0	5
Spain	1	0
Sweden	0	1
Switzerland	2	0
United Kingdom	9	7
United States	86	78
Uruguay	0	1

Table 7: Summary Statistics: (experiment_language)

Freq	(o)	(r)
Arabic	1	1
Dutch	5	7
English	101	96
France	1	0
French	3	0
German	13	23
Hebrew	3	0
Italian	2	3
Polish	1	0
Spanish	1	1

Table 8: Summary Statistics: (compensation)

Freq	(o)	(r)
cash	47	52
credit	57	58
mixed	18	15
nothing	9	6

Table 9: Summary Statistics: (subjects)

Freq	(o)	(r)
anyone	9	6
community	8	8
online	0	4
students	114	113

Table 10: Summary Statistics: (project)

Freq	
ee	18
ml1	13
ml3	10
rpp	90

Table 11: Summary Statistics: (seniority)

Freq	(o)	(r)
Assistant	0	14
Assistant Professor	2	22
Associate Professor	11	24
Professor	113	57
Researcher	5	14
