# Supplementary material for: Predicting the replicability of social science lab experiments
Source: PLoS One. 2019 Dec 5;14(12):e0225826. doi: 10.1371/journal.pone.0225826 (PMC6894796; doi:10.1371/journal.pone.0225826)
Supplement: S3 Table — Accuracy of prediction market data used in the paper. The ending prices for each asset are directly interpreted as replication probabilities and prediction accuracy is calculated based on a 50% probability cutoff. (PDF) [file pone.0225826.s003.pdf]

Table 12: Prediction Market Accuracy

	Full dataset	ML dataset
Pooled PM Accuracy:	66.7%	65.5%
RPP PM Accuracy:	69.2%	67.6%
EE PM Accuracy:	61.1%	61.1%
