# Supplementary material for: Predicting the replicability of social science lab experiments
Source: PLoS One. 2019 Dec 5;14(12):e0225826. doi: 10.1371/journal.pone.0225826 (PMC6894796; doi:10.1371/journal.pone.0225826)
Supplement: S4 Table — The contribution of all 15 principal components. Contributed variance tapers off quite slowly, indicating the lack of any strong linear structure. (PDF) [file pone.0225826.s004.pdf]

	s.d.	Var. \%	Cumulative
PC1	1.898	0.240	0.240
PC2	1.515	0.153	0.393
PC3	1.436	0.137	0.531
PC4	1.185	0.094	0.624
PC5	1.031	0.071	0.695
PC6	1.005	0.067	0.762
PC7	0.932	0.058	0.820
PC8	0.900	0.054	0.874
PC9	0.825	0.045	0.920
PC10	0.732	0.036	0.956
PC11	0.615	0.025	0.981
PC12	0.407	0.011	0.992
PC13	0.282	0.005	0.997
PC14	0.176	0.002	0.999
PC15	0.109	0.001	1.000

Table 13: PCA
