# Supplementary material for: Predicting the replicability of social science lab experiments
Source: PLoS One. 2019 Dec 5;14(12):e0225826. doi: 10.1371/journal.pone.0225826 (PMC6894796; doi:10.1371/journal.pone.0225826)
Supplement: S1 Text — (PDF) [file pone.0225826.s005.pdf]

For most variables we abstain from any sort of imputation, dropping the observation if we were not able to collect all data. However for cases when the planned sample size has not been imported, and thus the planned power cannot be calculated, we do estimate it using actual replication sample size as a proxy.

Since the ML data is pooled, whenever there is a lab-specific variation we use the modal option. If one study was replicated by three French, one Italian and two American labs, we mark the study as having been conducted in France.

Last, while the publication year of the original article is an ex ante relevant correlate of reproducibility, we have chosen to remove it from the training data since it also captures a feature of the paper selection process used in the ML projects. The authors wanted a benchmark for comparison, and included a number of papers that had been successfully replicated many times before. These studies are all older, making the publication year variable a proxy for papers with (already known) high replication rates.

Citations for papers and authors changes constantly. We downloaded citation counts in September 2016. These could easily be updated in future versions of the model.

Post-hoc power was calculated by the authors based on the hypothesis tests, effect sizes, and sample sizes used in the original experiment.
