# Supplementary material for: Predicting the replicability of social science lab experiments
Source: PLoS One. 2019 Dec 5;14(12):e0225826. doi: 10.1371/journal.pone.0225826 (PMC6894796; doi:10.1371/journal.pone.0225826)
Supplement: S2 Text — Discussion about the theoretical upper bound of prediction accuracy. (PDF) [file pone.0225826.s006.pdf]

Statistical inference and estimation are designed to make conclusions from noisy data, but this also necessarily implies that such conclusions will sometimes be wrong. Predicting the outcome of these conclusions, if the noise is indeed random, can thus never become perfect.

For example, when predicting the outcome of a hypothesis test with statistical power at 90%, one out of 10 true effects will not replicate. Similarly, a significance level of 5% will mean that one in 20 null effects will still be deemed significant. To see this more clearly, see the table below.

A prediction can be correct or not in different ways. However, even when the prediction is correct, it does not necessarily mean that the actual effect exists in the population. Instead, it could have been generated by the inherent noisiness of sampled data through Type I and Type II errors. When running replications, we think of these errors as bad scientific luck, and their presence is a reason why multiple replications by independent labs are helpful. But for our purposes, by definition, they make perfect predictions impossible.

Assuming that the noise we see is completely unpredictable, the best a perfect classification model could do is to always predict the experiment to replicate when the null is false, and not otherwise. Such a model is what we think should be considered the upper bound to which our results can be compared.

So how should we estimate this bound? A perfect model that always predicts the true state would still have an error rate of 1-Power for true effects and $\alpha = 5\%$ for null effects. To know the accuracy of a perfect model, we thus need the proportion of true effects to null effects in our set of studies, and also the power of all tests of true effects. Most replications in our data set aimed at being able to detect the effect of the original study with 90% power. But since effect sizes are almost always smaller than what was initially claimed, real power lies below 90% in most of the replications. With a significance rate of 5%, while we do not know the true proportion of null effects, even a rather conservative measure would put the true upper bound on accuracy somewhere below 90%.

A similar argument can be made for the continuous model. Each replication is a noisy sample of observations from which we calculate the mean and the standard deviation of the effect size as to approximate population parameters.

Without knowing the population parameters, we cannot say much about the size of the error made in the replication. But since this sampling error is random it cannot be predicted by the model. Any relative effect size estimate prediction will just like the prediction of binary replication never be perfect. The error rate is bounded from below.

Finding these theoretical bounds is beyond the scope of this paper. They are functions of unknown parameters and therefor not informative in finding the actual bounds of the model as implemented here. Further theoretical research into the limits to replication prediction is a welcome avenue for future research.

		Outcome of replication	
		Not replicate	Replicate
Prediction	Not replicate	True Negative	False Negative
	Replicate	False Positive	True Positive

		Outcome of replication	
		Not replicate	Replicate
True State	H0 True	Correct, $1 - \alpha$	Type I Error, α
	H0 False	Type II Error, β	Power, $1 - \beta$
